# A small cassette enables conditional gene inactivation by CRISPR/Cas9

**DOI:** 10.1038/s41598-017-16931-z

**Published:** 2017-12-01

**Authors:** Paloma M. Guzzardo, Christina Rashkova, Rodrigo L. dos Santos, Raha Tehrani, Philippe Collin, Tilmann Bürckstümmer

**Affiliations:** 1 Horizon Genomics GmbH, Vienna, Austria; 2 Horizon Discovery Ltd, Cambridge, UK

## Abstract

The availability of CRISPR/Cas9 technology has enabled the rapid establishment of gene knockouts in many cell types and even whole organisms. However, conditional inactivation of essential genes remains a challenge. We devised an approach named DECAI (DEgradation based on Cre-regulated- Artificial Intron). It utilizes a small cassette of just 201 nucleotides that is inserted into the coding exon of a target gene using CRISPR/Cas9 technology and homology-directed repair. As its sequence is derived from an artificial intron, the cassette is removed by the splicing machinery and thus leaves no trace in the “off-state”. Upon activation with Cre recombinase (“on-state”), the intron is crippled and the target gene is disrupted by a series of stop codons. We exemplify the utility of this approach on several non-essential and essential human genes. Clones bearing the conditional knockout cassette are recovered at frequencies above 5% and cassette function can be traced at the genomic DNA and the mRNA level. Importantly, cassette activation leads to loss of gene expression as judged by flow cytometry, Western blot or immunofluorescence. Altogether, this highlights the broad utility of the approach for conditional gene inactivation and suggests that this tool could be used to study the loss-of-function phenotypes of essential genes.

## Introduction

Genome editing has been revolutionized by the discovery of CRISPR/Cas technology which enables the targeted introduction of double strand breaks into the human genome with an unprecedented precision and efficiency^1–4^. Once the double-strand break has been established, the cell will repair the break by one of two common pathways: in the presence of a homology template, the cell will use homology-directed repair or homologous recombination^5^. In the absence of homologous sequences, the cell will employ non-homologous end joining to repair the break^6^.

As non-homologous end joining is by nature quite imprecise, it triggers the accumulation of small insertions or deletions (so called indels)^6^. If such indels lie within the coding sequence of a gene, they often disrupt the open reading frame and thus abrogate the translation (or transcription; as a result of nonsense-mediated decay) of the corresponding gene. This has been exploited to create cell lines or whole organisms (e.g. mice) bearing so called gene knockouts. Gene knockouts have been instrumental for studying loss-of-function phenotypes^7^.

However, some human genes are essential and hence, the corresponding gene knockouts are lethal^8^. By definition, essential genes are key regulators of cellular processes and, not surprisingly, many of them represent important drug targets. As a consequence, there is a large unmet need for tools to facilitate the study of essential genes.

Historically, the function of essential genes has been explored using conditional gene knockouts. One of the most common strategies used to generate conditional knockouts is based on the site-specific recombinase technology, specifically the Cre-loxP system. This system utilizes Cre recombinase which will trigger recombination between two loxP sites upon recognition. To generate conditional knockouts using this strategy, one would first have to flank the target gene or an exon of this gene with loxP sites that enable Cre recombinase-mediated deletion^9^. This would involve the assembly of large homology donors, which could be costly and difficult to synthesize. These donors would then be used to establish genetically engineered cells by spontaneous homologous recombination (e.g. in mouse embryonic stem cells) or, more recently, by CRISPR-assisted homologous recombination. Unfortunately, none of these approaches are efficient and both require the screening of hundreds to thousands of clones by Southern blotting or PCR. These modified cells could then be used to create transgenic mice, which in combination with a tissue- or cell-specific promoter driven Cre can be used to study the function of essential genes. It is important to highlight that this method depends on all alleles of the target gene containing the loxP sites in order to disrupt gene expression efficiently upon Cre recombination. In *in-vivo* models this is achieved by breeding mice which are heterozygous for the modification. In cell lines, however, it would be necessary to screen large numbers of clones until a bi-allelically modified clone is identified or to perform sequential rounds of editing.

Very recently, a conditional knockout approach known as CRISPR-FLIP was introduced^10^. It is based on an invertible intronic cassette (FLIP), similar to COIN^11^. In the non-mutagenic orientation, the FLIP cassette expresses the puromycin resistance gene to select for correct nuclease-assisted targeting into the exon of one allele. Upon exposure to Cre recombinase the FLIP cassette is inverted to a mutagenic configuration that activates a cryptic splice acceptor and polyadenylation signal and disrupts the initial splice acceptor resulting in the loss of gene function. While this system is attractive, it has several shortcomings: (i) the cassette that is used as a donor is large (1,931 bp) and hence, the assembly of targeting constructs with homology arms is costly, cumbersome and time-consuming; (ii) the cassette contains an antibiotic resistance gene which is not always desired and (iii) the expression of the gene of interest is disrupted by a splice acceptor which can be leaky.

In this report, we aimed to devise an approach that allows the generation of conditional gene knockouts on a large scale with the following features: (i) engineering should be straightforward in diploid or even polyploid cells; (ii) the approach should not require any prior insight into the structure/function of the targeted gene product; (iii) in the “off-state” of the cassette, the activity of the target gene product should be largely unaffected; (iv) cassette activation (transition to “on-state”) should trigger a profound decrease in gene expression. Based on these requirements, we designed a genetic approach named DECAI (DEgradation based on Cre-regulated Artificial Intron), where conditional gene knockouts can be generated by inserting a small intron cassette into the coding sequence of an exon.

## Materials and Methods

### Cassette sequences

Five different cassettes were tested with varying locations of the loxP sites within the synthetic intron sequences (Table 1). For NanoLuc trials, a ‘recombined’ version of each cassette, with the predicted sequence after treatment with Cre recombinase, was tested alongside. Important features of the cassettes are highlighted: 5′ and 3′ splice sites are shown in bold, the branch point is double underlined and the polypyrimidine tract is underlined. loxP sites are in lower case letters and the stop codons in all three reading frames are shown in italics.

**Table 1:** Sequences of five cassette variants.

Name	Cassette sequence	Cassette sequence after Cre recombination
Variant 1	**G** ***TAA*** **G**TATCAAGGT*TAG*AAGACAGGTT*TAA*GGAGACCAATAGAAACTGGGCTTataacttcgtatagcatacattatacgaagttatGTCGAGACAGAGAAGACTCTTGCGTTTCTGATAGGCACataacttcgtatagcatacattatacgaagttatCTATTGGTCTTACTGACATCCACTTTGCCTTTCTCTCCA **CAG**	**G** ***TAA*** **G**TATCAAGGT*TAG*AAGACAGGTT*TAA*GGAGACCAATAGAAACTGGGCTTataacttcgtatagcatacattatacgaagttatCTATTGGTCTTACTGACATCCACTTTGCCTTTCTCTCCA **CAG**
Variant 2	**G** ***TAA*** **G** *TAa*taacttcgta*tag*catacattatacgaagttatTCAAGGTTAGAAGACAGGTTTAAGGAGACCAATAGAAACTGGGCTTGTCGAGACAGAGAAGACTCTTGCGTTTCTGATAGGCACataacttcgtatagcatacattatacgaagttatCTATTGGTCTTACTGACATCCACTTTGCCTTTCTCTCCA **CAG**	**G** ***TAA*** **G** *TAa*taacttcgta*tag*catacattatacgaagttatCTATTGGTCTTACTGACATCCACTTTGCCTTTCTCTCCA **CAG**
Variant 3	**G** ***TAA*** **G** *TAat*aacttcgta*tag*catacattatacgaagttatTCAAGGTTAGAAGACAGGTTTAAGGAGACCAATAGAAACTGGGCTTGTCGAGACAGAGAAGACTCTTGCGTTTCTGATAGGCACCTATTGGTCTTACTGACATCCACTTTGCCTTTCTCTCCAataacttcgtatagcatacattatacgaagttat**CAG**	**G** ***TAA*** **G** *TAa*taacttcgta*tag*catacattatacgaagttat**CAG**
Variant 4	**G** ***TAA*** **G** *TAa*taacttcgta*tagc*atacattatacgaagttatTCAAGGTTAGAAGACAGGTTTAAGGAGACCAATAGAAACTGGGCTTGTCGAGACAGAGAAGACTCTTGCGTTTCTGATAGGCACCTATTGGTCTTACTGACATCCACTTTGCCataacttcgtatagcatacattatacgaagttatTTTCTCTCCA **CAG**	**G** ***TAA*** **G** *TAa*taacttcgta*tag*catacattatacgaagttatTTTCTCTCCA **CAG**
Variant 5	**G** ***TAA*** **G** *TAa*taacttcgta*tag*catacattatacgaagttatTCAAGGTTAGAAGACAGGTTTAAGGAGACCAATAGAAACTGGGCTTGTCGAGACAGAGAAGACTCTTGCGTTTCTGATAGGCACCTATTGGTCTTACTGACAataacttcgtatagcatacattatacgaagttatTCCACTTTGCCTTTCTCTCCA **CAG**	**G** ***TAAG*** *TAa*taacttcgta*tagc*atacattatacgaagttatTCCACTTTGCCTTTCTCTCCA **CAG**

The artificial intron was modified to contain a series of stop codons as well as loxP sites. The main difference between the five variants is the location of the loxP sites.

### gRNA sequences

The following gRNA sequences were used to insert the artificial intron cassette (Table 2).

**Table 2:** Sequences of the gRNAs utilized in this study.

Targeted gene	gRNA sequence
CD46	GATAAGGGTTTTTACCTCGA
METTL16	TCTGACGTGTACTCTCCTAA
Oct4/ POU5F1 site#1	GCACTAGCCCCACTCCAACC
Oct4/ POU5F1 site#2	ACCACCTGGAGGGGGCGAGA

The table depicts the various genes that were targeted and the corresponding gRNA sequences.

### NanoLuc assay

HAP1 or HEK293T cells were transfected with a pcDNA3.1 plasmid encoding NanoLuc or NanoLuc containing artificial intron variants (see Table 1) and pGL4.53[luc2/PGK] vector (Promega) for normalization. Cells were harvested 24–48 h post transfection and analysed using the Nano-Glo Dual Luciferase Assay System (Promega) according to manufacturer’s instructions.

### Generation of cell lines

HAP1 cells were transfected with TurboFectin transfection reagent (OriGene). In brief, 0.8 × 10^6^ cells were seeded in a 6-well plate and transfected on the following day with 1.5 µg of Cas9 expression plasmid (#48137 from Addgene), 1 µg of U6 driven gRNA expression plasmid (Horizon Discovery), 0.5 µg of PCR product encoding the homology donor and 0.2 µg of plasmid encoding a blasticidin resistance gene. 24 h post transfection, cells were treated with 20 µg/ml of blasticidin for 24 h to eliminate untransfected cells. Single clones were obtained by limiting dilution. Positive clones were then identified by PCR screening.

iPS cells cultured in TeSR-E8 and Vitronectin XF (both STEMCELL Technologies) were transfected with the P3 Primary Cell solution using the CM-138 pulse in the 4D-Nucleofector (Lonza). In brief, 1.0 × 10^6^ cells were transfected with 2.5 µg of Cas9 expression plasmid, 2.5 µg of gRNA expression plasmid, 2.5 µg of plasmid encoding the donor and 0.5 µg of plasmid encoding a blasticidin resistance gene. Cells were plated in culture media supplemented with 10 µM ROCKi (Y-27632, Abcam) for 24 h, and then treated with 10 µg/ml of blasticidin for 24 h to eliminate untransfected cells. Recovered colonies were single cell plated after treatment with StemPro Accutase (Thermo Fisher Scientific) and clones were manually picked into 96 wells for screening.

### Genomic DNA isolation and PCR

Genomic DNA was isolated using the QIAamp DNA Mini Kit (Qiagen) according to manufacturer’s instructions. PCRs were done with the primers below using the GoTaq DNA Polymerase (Promega) following manufacturer’s instructions.

The following primers were used for PCR (Table 3).

**Table 3:** Primers used for PCR-based genotyping.

Name	Forward primer	Reverse primer
METTL16-DECAI genomic DNA	TTCTGCCTGTTTGCCGTAGA	TTGTCAGAATCCTGGTGACCG
CD46-DECAI genomic DNA	TGCATTCCATTCCTTGTCTCTG	AAGACACTTTGGAACTGGGGG
Oct4-DECAI#1	CTCTGAGGTGTGGGGGATT	TGCTCCAGCTTCTCCTTCTC
Oct4-DECAI#2	GACACCTGGCTTCGGATTT	CCCCACAGAACTCATACGG

Clones obtained from each targeting event were analysed by PCR from genomic DNA using the primers depicted above.

### RNA isolation, cDNA synthesis and qRT-PCR

Total RNA was isolated from cells using the RNeasy mini kit (QIAGEN) in accordance with manufacturer’s protocol. For RNA from Hap1 cells, 1 µg of total RNA was reverse transcribed using oligo dT primer and M-MLV Reverse Transcriptase (Promega). The cDNA was then used as a template for PCR using the primers listed in Table 4. For RNA from iPSCs, 1 µg of total RNA was reverse transcribed into cDNA using the SuperScript III First-Strand Synthesis SuperMix kit (Thermo Fisher Scientific). 10 ng of cDNA was used for qRT-PCR reactions that were set up in triplicates using either TaqMan Gene Expression Master Mix (Thermo Fisher Scientific). TaqMan gene expression assays (Thermo Fisher Scientific) were used for each gene analysed. qRT-PCR experiments were performed using QuantStudio 6 Flex Real Time PCR System (Thermo Fisher Scientific). All values were normalized to an internal control (GAPDH) and brought to power of 2. Each target’s value was then compared to the highest value of that target across all samples. All samples were done in technical triplicates and the median of these is displayed. The following TaqMan gene expression assays were used: Gapdh (Hs03929097_g1), Oct4 (Hs00999632_g1), Nanog (Hs04399610_g1), Sox2 (Hs01053049_s1), Cdx2 (Hs01078080_m1), Eomes (Hs00172872_m1) (all from Thermo Fisher Scientific).

**Table 4:** Primer used for RT-PCR.

Name	Forward primer	Reverse primer
CD46-DECAI RT-PCR	GGTCAAATGTCGATTTCCAGTAGT	GACACTTTGGAACTGGGGGA
GAPDH RT-PCR	GAAGGTGAAGGTCGGAGT	GAAGATGGTGATGGGATTTC

CD46 mRNA was reverse transcribed using an oligo dT primer and cDNA was analysed by RT-PCR using the primers specified above. GAPDH was amplified as a reference.

The following primers were used for RT-PCR (Table 4).

### Retroviral production and transduction of cells

Approximately 1 × 10^7^ HEK293T cells were transfected with 1.7 µg pAdvantage plasmid (Promega), 2.6 µg VSV-G expression vector, 4 µg gag-pol expression vector and 6.6 µg CreERT2 expression vector, using TurboFectin transfection reagent (OriGene). Medium was changed 24 hours after transfection. Viral supernatant was collected 48 and 72 hours after transfection, and used to transduce the METTL16-DECAI cells. Since the Cre-ERT2 retrovirus also encoded a PGK-PuroR cassette, cells containing Cre could be enriched for by selecting with 0.3 µg/ml puromycin.

### Flow cytometry, Western Blotting and Immunocytochemistry

For the HAP1 experiments, cells were trypsinized and washed with PBS. Cells were stained with a CD46-specific antibody (APC-CD46; catalogue #564253; BD Biosciences) in FACS buffer (5% FCS in PBS) for 30 minutes and excess antibody was removed by washing with PBS. Cells were analysed by flow cytometry using the BDLSR Fortessa.

For the human iPS cell experiments, cells were fixed and permeabilized using the Cytofix/Cytoperm kit (BD) according to manufacturer’s instructions. Cells were then stained using the following directly conjugated antibodies: Alexa Fluor® 488 anti-Oct4 (653706, BioLegend) and PE/Cy7 anti-human SSEA-4 (330420, BioLegend). Cells were analysed using the iQue Screener PLUS (IntelliCyt). Data was analysed using the FlowJo software.

For Western blotting, lysates were prepared using Frackelton buffer (10 mM Tris/HCl pH 7.5, 50 mM NaCl, 30 mM sodium pyrophosphate, 1% Triton X-100, 50 mM NaF and protease inhibitors). Lysates were separated on 10% SDS-PAGE and blotted on Nitrocellulose membranes. Membranes were stained with a primary antibody (anti-CD46, ab108307, Abcam) and a secondary antibody (anti-rabbit, 111-035-003, Jackson Immunoresearch Europe) and visualized using ECL reagent (Thermo Fisher Scientific).

For Immunocytochemistry, the PSC 4-Marker Immunocytochemistry Kit (Thermo Fisher Scientific, A24881) was used. Briefly, iPS cells were fixed using 4% PFA for 15 min, permeabilized using 1% Saponin for 15 min, and blocked with 3% BSA for 30 min (all at room temperature). Primary antibodies used: rabbit anti-Oct4 (A24867) and mouse IgG3 anti-SSEA4 (A24866); secondary antibodies used: Alexa Fluor 594 donkey anti-rabbit (A24870) and Alexa Fluor 188 goat anti-mouse IgG3 (A24877). DAPI was used for nuclear staining.

### Data availability

All data generated or analysed during this study are included in this published article (and its Supplementary Information files).

## Results

**Figure 1:**
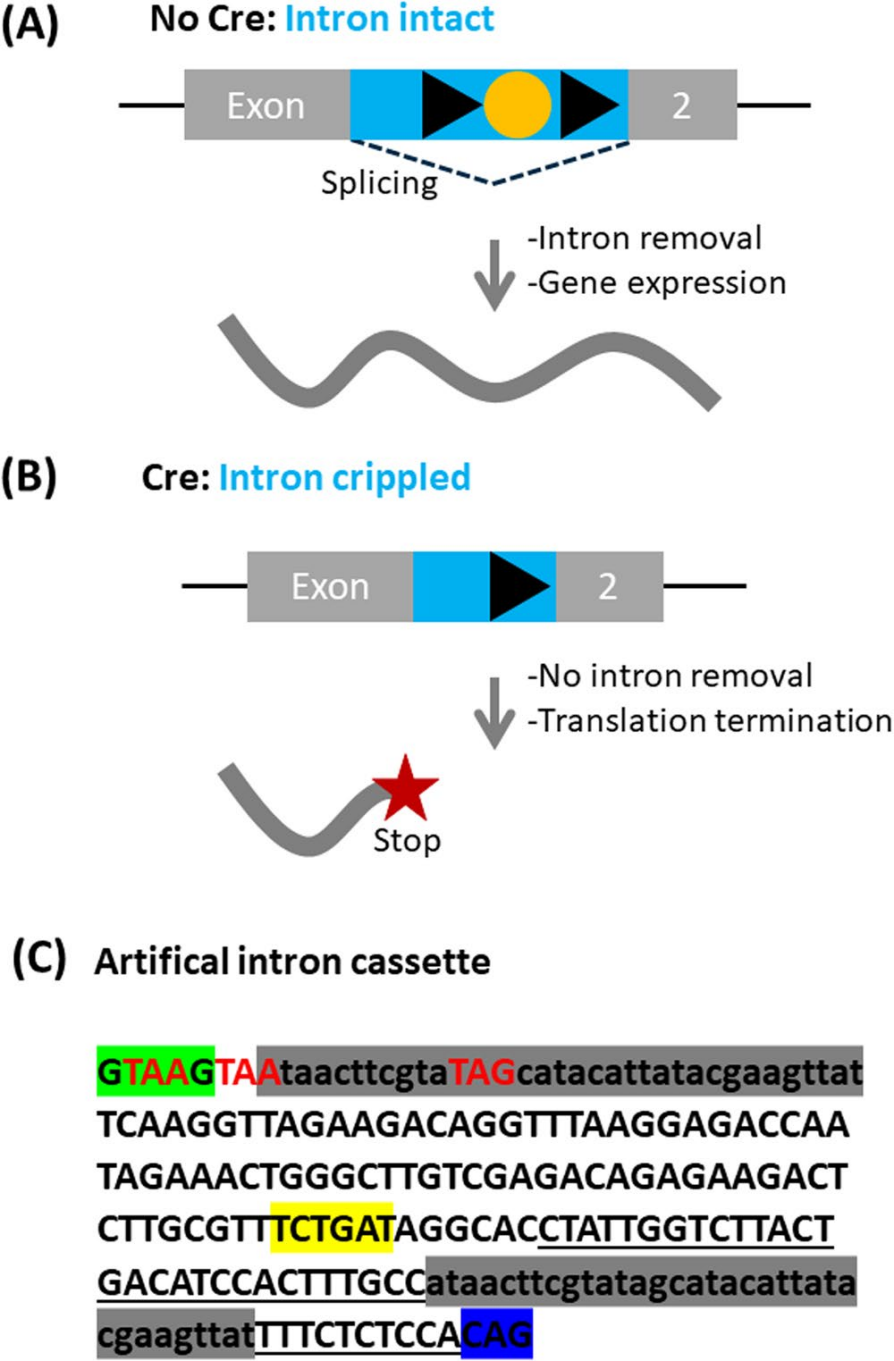
Approach for conditional gene inactivation using artificial introns. (**A**) For the purpose of conditional gene inactivation, an artificial intron is introduced into a coding exon of a gene. In the artificial intron (marked in blue), the branch point (in yellow) is flanked by two parallel loxP sites (black triangles). Under steady-state conditions, the intron gets removed by splicing and leaves the mRNA of the target gene intact. (**B**) Upon recombination with Cre, the branch point is excised and the intron is inactivated. Consequently, the intron is no longer removed and the ribosome reads through the intronic sequence, running into one of three stop codons that were added to the artificial intron cassette. (**C**) DNA sequence of the artificial intron that was used for most of the experiments presented in this manuscript. Important features are highlighted: splice donor in green, loxP sites in grey, stop codons in three reading frames in red, branch point in yellow, polypyrimidine tract is underlined and splice acceptor in blue.

To generate conditional gene knockouts, we designed a genetic approach that was based on inserting a small intron cassette into the coding sequence of an exon. Introns were favoured because they are removed by splicing and thus leave the targeted gene intact until the cassette is activated (Fig. 1A). To overcome limitations of previous similar approaches, we decided to minimize the size of the intron and to base our design on a very small chimeric intron. Introns contain three elements that are critical for their function: the splice donor, the branch point and the splice acceptor. We reasoned that removal of the branch point by Cre/loxP recombination could cripple the intron, thus triggering the translation of truncated intronic sequence (Fig. 1B). Addition of three stop codons (one for each reading frame) would then lead to translational termination of the gene and would thus abrogate gene expression.

Since the intron we used is very small, the precise architecture of the cassette was not straightforward and the two major risks included: (i) cassette modification could disturb intron splicing, hence leaving the cassette in a constant “on-state”; (ii) as the branch point sequence is quite degenerate, nearby sequences may take over branch point function upon its removal.

To overcome these difficulties, we established an assay based on NanoLuc reporter gene expression where we could easily test a variety of cassette configurations. A series of NanoLuc expression plasmids were created in which the different cassette designs were inserted into one specific location of the NanoLuc sequence. We also included the respective sequences as they would occur after Cre recombination, thus simulating the Cre/loxP excision event. An ideal cassette leaves NanoLuc expression unaffected (“off-state”) but triggers a profound decrease upon Cre/loxP recombination (“on-state”).

**Figure 2:**
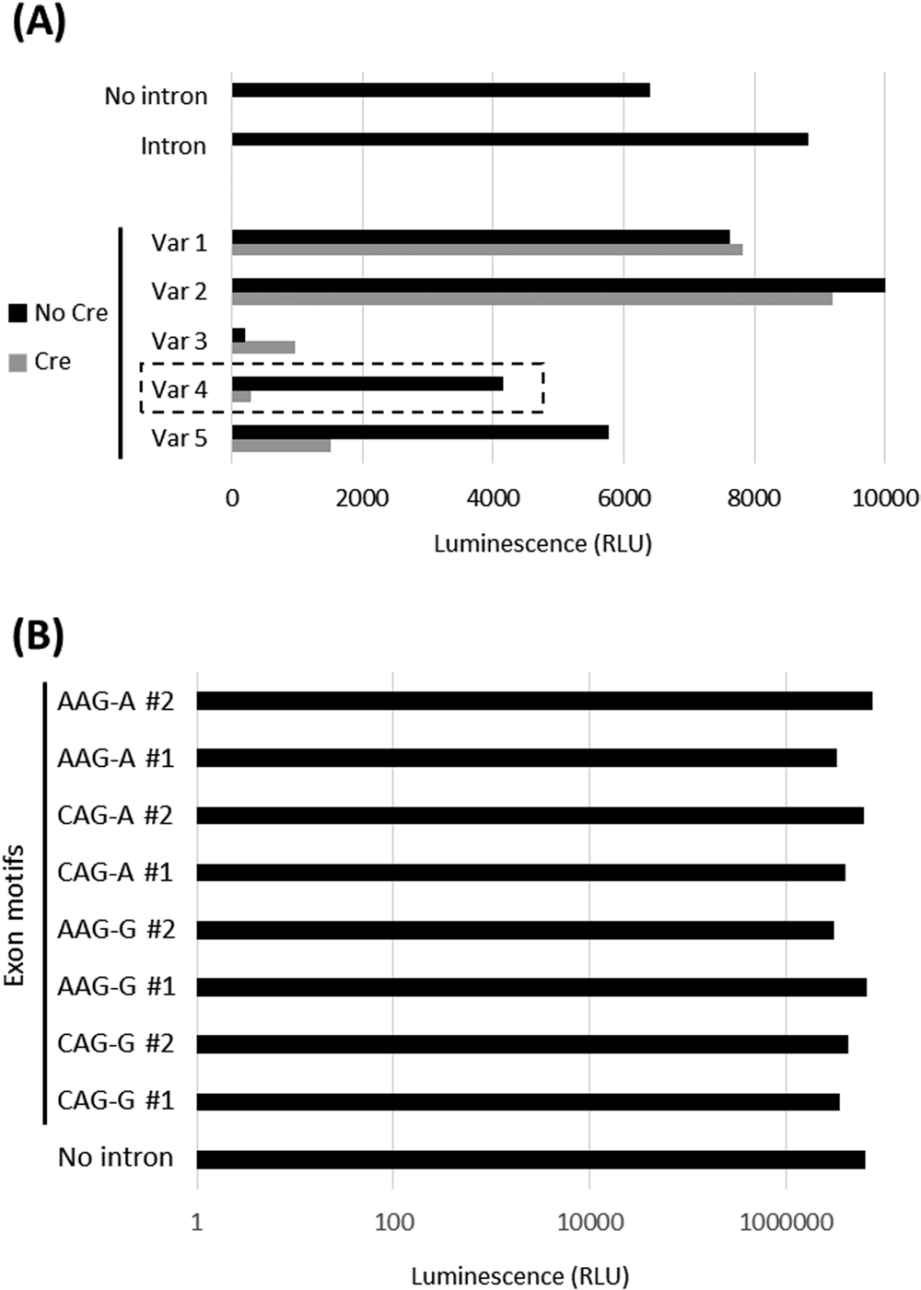
Identification of the artificial intron configuration that enables conditional gene inactivation. (**A**) HAP1 cells were transfected with NanoLuc without intron (“No intron”) or one harbouring the intron without further modification (“Intron”). In addition, a set of cassette variants (Var 1–5) were tested in which loxP sites positioning relative to the branch point varied. Note that we included two designs for each cassette, one in which the cassette was intact (“no Cre”) and one in which the sequence between the loxP sites had been removed (“Cre”). NanoLuc levels were measured 24 h post transfection using the Nano-Glo Dual assay. (**B**) Variant 4 identified in Fig. 2A was placed into various sequence contexts within the NanoLuc gene, reflecting one of four possible insertion sites: CAG-G, AAG-G, CAG-A and AAG-A (where the hyphen denotes the site where the intron got inserted). For each of these, two sites were evaluated. HEK293T cells were transiently transfected with these various constructs and NanoLuc luciferase activity was measured using the NanoGlo Dual assay 48 h hours after transfection.

First, we noted that insertion of the intronic sequence without modification (no loxP sites) was well tolerated (Fig. 2A). This suggested that the intron was functional in principal and that the site within NanoLuc tolerated an intronic insertion. Next, we assessed the various variants side-by-side (Fig. 2A). Of interest, introduction of cassette variant 3 lead to a strong decrease in NanoLuc expression even in the “off-state” of the cassette, suggesting that this cassette modification was incompatible with proper intron splicing. Variants 1 and 2 were well tolerated, yet we did not observe any reduction in gene expression upon Cre recombination, suggesting that alternative branch points had taken over. Of the variants tested, variant 4 (Fig. 1C) was most fit-for-purpose as it was well tolerated in the “off-state” and triggered a profound decrease (~14 fold) in NanoLuc expression upon cassette activation.

Splicing is strongly dependent on the sequence context and requires certain specific sequences at the intron/exon boundary. Introns of the GT-AG category, such as the one we chose, have a preference for CAG or AAG upstream of the splice donor and G or A downstream of the splice acceptor^12^. Hence, the choice of target sites to insert an intron into the coding sequence of an exon should be restricted to CAG-G, CAG-A, AAG-G or AAG-A (where the hyphen denotes the site where the intron is inserted). To test whether all four sites tolerated the insertion of the intron equally, we created eight NanoLuc constructs, targeting eight different intron insertion sites (two for each of the four motifs). Transient overexpression in HEK293T suggests that all of these sites tolerated the insertion of an intron equally (Fig. 2B). This suggests that our approach is broadly applicable across the various insertion sites depicted above.

Having established a cassette in which the desired features had been encoded in just 201 nucleotides, we decided to test this cassette in human cells. In order to introduce the intron, we designed and synthesized homology donors of ~1 kb in which the cassette had been placed in the middle (leaving ~400 bp homology arms on either end; Supplementary Figure 1). We targeted exons of two human genes: (i) CD46 because it is cell-surface expressed and can be easily detected by FACS; and (ii) METTL16 because it is an essential gene and cassette activation should trigger cell death.

We chose the human haploid cell line HAP1 for the ensuing targeting experiments because these cells contain a single copy of every human gene and conditional gene inactivation is thus straightforward^13^. We chose exons that were at least 100 bp from the start codon and within the first 30% of the coding sequence of the gene. Following transfection of HAP1 cells with Cas9, the respective gene-specific gRNA and the homology donor, we obtained clonal cell lines by limiting dilution. Single clones were genotyped by PCR to identify clones in which cassette integration had occurred. Note that we did not select for cassette integration as the conditional KO cassette itself does not contain any selectable marker. In spite of this, we obtained targeted clones at frequencies of 5% or higher (Table 5). Of interest, the frequency at which the cassette was incorporated was higher in the essential gene (METTL16) than in the non-essential gene (CD46). While this may well be a coincidence, it is plausible to assume higher targeting frequencies for essential genes as other editing events (e.g. the accumulation of indels) may be subject to negative selection.

**Table 5:** Frequency of cassette integration by homology-directed repair.

Gene	Cell line	# of clones screened	# of clones containing the cassette
CD46	HAP1	96	8
METTL16	HAP1	96	32
OCT4 site#1	iPSC	56	4 (2 KI/KI, 1 KI/WT, 1 KI/indel)
OCT4 site#2	iPSC	56	3 (1 KI/KI, 2 KI/indel)

HAP1 cells or human iPSCs were targeted with Cas9 and gRNAs specific for certain human genes along with homology donors specifying the artificial intron cassette. The table depicts the number of clones that were screened by PCR and the number of clones that were retrieved containing a cassette integration event.

**Figure 3:**
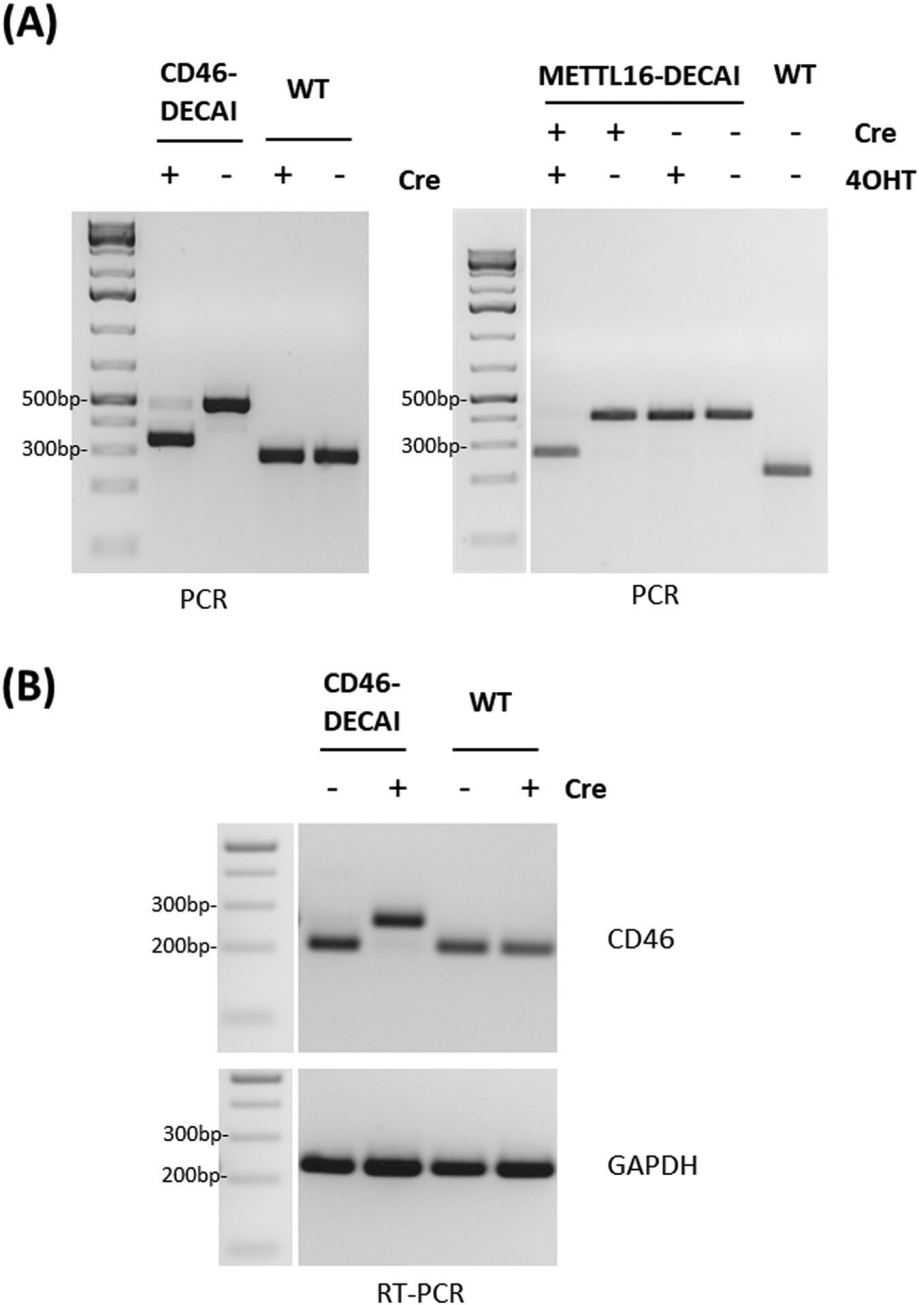
Cre-recombination leads to branch point excision. (**A**) Genomic DNA was isolated from cells transfected with Cre (for CD46) or cells transduced with Cre-ERT2 and treated with 4-hydroxitamoxifen (4-OHT; for METTL16). Samples were genotyped using PCR primers specific for CD46 or METTL16. (**B**) mRNA was isolated from cells expressing Cre, reverse transcribed using oligo (dT) and analysed with primers specific for the CD46 cDNA.

Clonal cell lines bearing the cassette were expanded and Cre recombinase was expressed to trigger cassette activation. For CD46, HAP1 cells were transiently transfected with a plasmid encoding Cre. For METTL16, cells were infected with a retrovirus encoding Cre-ERT2 recombinase. Cre-ERT2 was activated by addition of 4-hydroxitamoxifen (4-OHT). Genomic DNA from the resulting cell lines was isolated and analysed by PCR (Fig. 3A). Cell lines bearing tagged alleles displayed a band that was shifted in size by ~200 bp, corresponding to the artificial intron that had been integrated at the targeted site. Following transduction with Cre (and activation with 4-OHT), this band was shifted to smaller size by ~150 bp, corresponding to the sequence that had been removed by Cre recombination. This indicates that Cre-mediated recombination faithfully triggers the removal of the sequences between the two parallel loxP sites. We also isolated mRNA from the cell line in which CD46 had been tagged with the artificial intron and analysed the CD46 transcript by RT-PCR (Fig. 3B). In the absence of Cre, the intron was efficiently removed in the CD46-DECAI clone and thus not detectable at the mRNA level. Upon addition of Cre, the intron was inactivated and the crippled intronic sequence was incorporated in the mRNA sequence, leading to a shift towards higher molecular weight.

**Figure 4:**
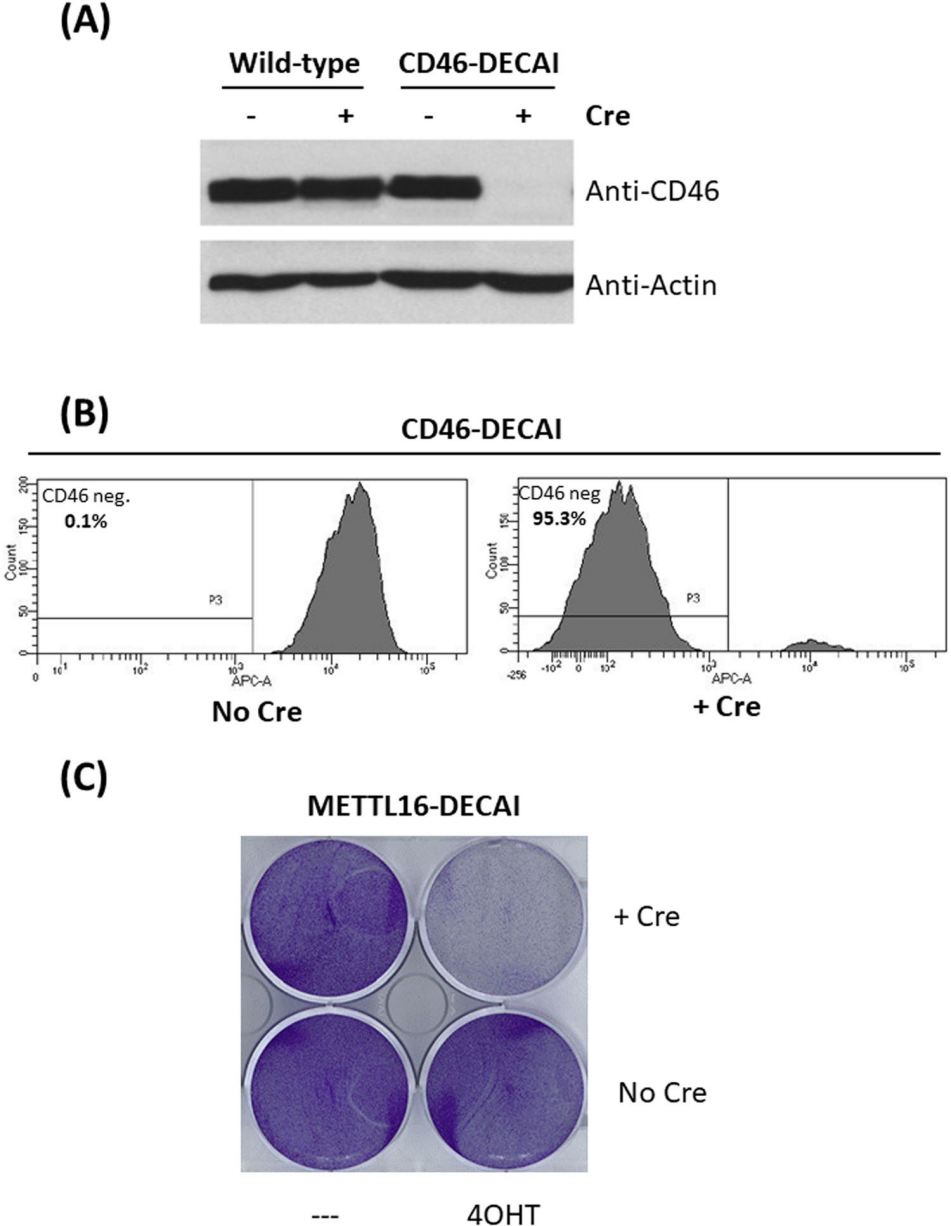
Artificial intron cassette activation leads to conditional gene inactivation. (**A** and **B**) HAP1 cells bearing the artificial intron in CD46 were transfected with Cre recombinase. For Western blotting, cells were analysed using a CD46-specific antibody. For flow cytometry, cells were stained with a CD46-specific antibody. (**C**) METTL16-DECAI cells were transduced with Cre-ERT2, treated with 4-OHT as indicated and stained with Crystal Violet.

Next, we assessed the impact of cassette activation on gene expression and gene function. We started our analysis with CD46 as the gene is non-essential and its expression can be easily visualized. We compared cells bearing the intron to wild-type cells and noted no difference, neither by Western blotting (Fig. 4A) nor by flow cytometry (Supplementary Figure 2). This indicates that the artificial intron did not disrupt endogenous gene expression in the “off-state”. Upon transfection of cells with Cre, CD46-DECAI cells abrogated CD46 expression almost completely, whereas wild-type cells were unaffected (Fig. 4A and B). The few remaining CD46-positive cells (~5% as judged by flow cytometry) likely arose from incomplete transfection of cells with Cre recombinase.

We also assessed the impact of the artificial intron on the essential gene METTL16. Transduction of cells with Cre-ERT2 and activation of Cre-ERT2 with 4-OHT triggered cell death in METTL16-DECAI cells (Fig. 4C). We noted that cell death was incomplete (data not shown), suggesting that retroviral Cre-ERT2 expression may have been silenced in a subpopulation of cells. To overcome this silencing, we isolated single cell clones bearing retroviral insertions of Cre-ERT2 and activated Cre-ERT2 by 4-OHT treatment (Supplementary Figure 3). As expected, some clones showed complete cell death upon 4-OHT treatment, whereas others were almost unaffected. This highlights the need for effective delivery and expression of Cre.

We also wanted to apply our approach in a more physiological setting and decided to test it in human induced pluripotent stem cells (iPSCs). Human iPSCs are pluripotent and their pluripotency depends on the expression of the transcription factor Oct4/POU5F1^14^. Inactivation of Oct4 triggers the collapse of the core transcriptional network leading to rapid loss of pluripotency. This is associated with drastic changes in colony morphology and loss of other pluripotency markers, such as Nanog, Sox2 and SSEA4. We created homology donors for Oct4/POU5F1 (400 bp homology arms flanking the DECAI cassette) and targeted two independent sites in exon 1 (Supplementary Figure 1). Clonal cell lines were established and genotyped by PCR. Both homozygous and heterozygous cell lines were achieved (Table 5).

**Figure 5:**
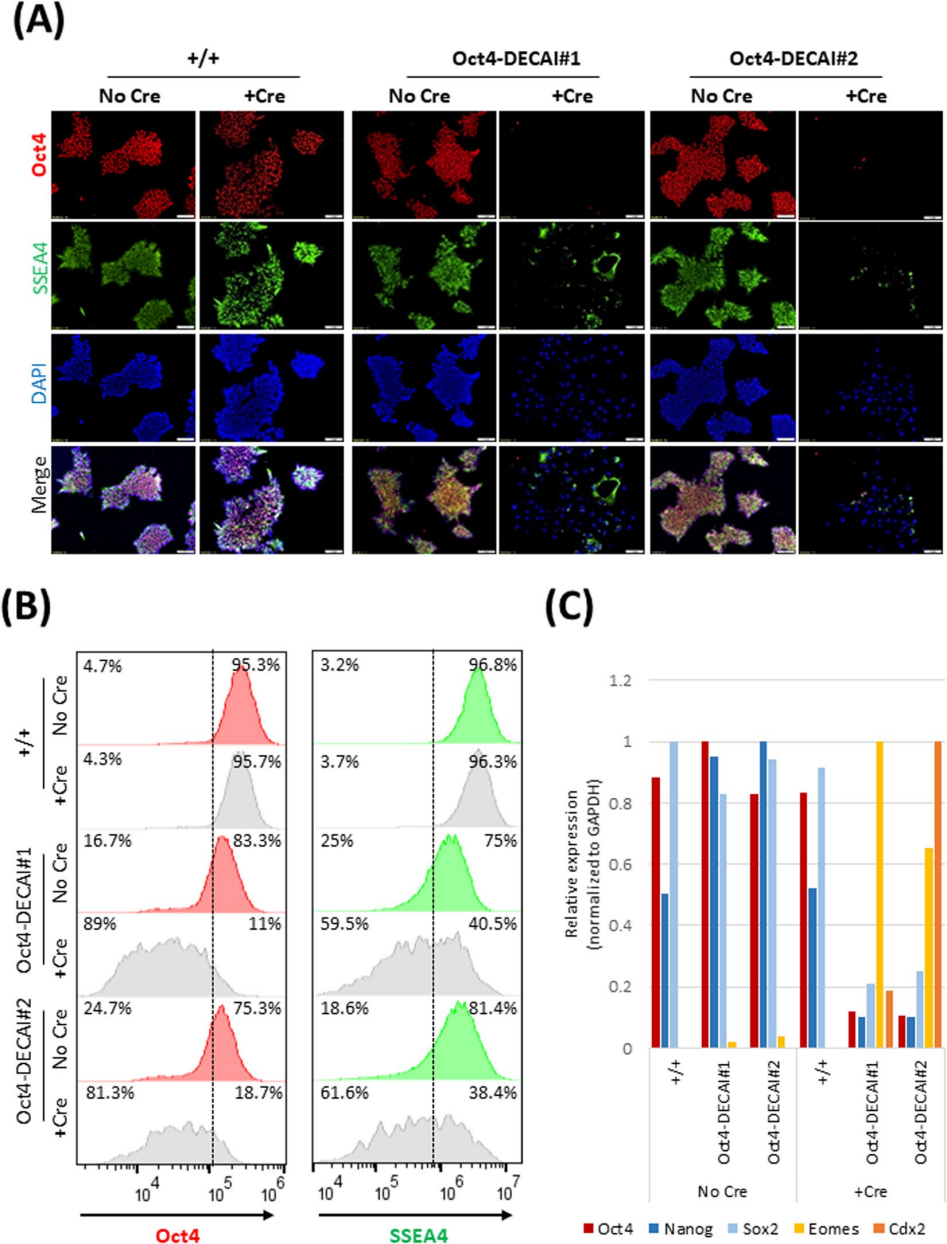
Generation of Oct4-conditional knockout human iPS cells. (**A**) Wild-type cells (+/+) or cells bearing the artificial intron in exon 1 of Oct4 (Oct4-DECAI #1 and Oct4-DECAI #2, created with two independent gRNAs) were transfected with Cre-ERT2 recombinase and 4-OHT or left untreated. Cells were fixed in 4% PFA and stained for Oct4 and SSEA4 using specific antibodies. DAPI was used for nuclear staining. Images were acquired with an Olympus IX83 microscope (10X magnification, 100 µm scale bars). For bright field images see Supplementary Figure 5. (**B**) Flow cytometry analysis of the cells before (No Cre) and after Cre treatment (+Cre) stained with Oct4 and SSEA4 specific antibodies. (**C**) qRT-PCR analysis was performed before and after induction of Cre recombination. Expression levels of Oct4, two additional pluripotency markers (Nanog and Sox2), and two differentiation markers (Cdx2 and Eomes) are shown. qRT-PCR values are normalized to GAPDH and shown as relative to the highest value of that target across all samples.

We selected two homozygous clones bearing the artificial intron in distinct genomic locations in exon 1 to study in greater detail. Immunofluorescence staining (Fig. 5A), flow cytometry analysis (Fig. 5B) and RNA expression (Fig. 5C) showed that human iPSC colonies bearing the intron expressed similar levels of Oct4 as non-targeted cells, indicating that the intron by itself did not affect Oct4 expression. Likewise, pluripotency was retained as suggested by the SSEA4 staining and normal expression of pluripotency markers Nanog and Sox2 (Fig. 5A–C). Upon recombination with Cre, wild-type cells were essentially unaffected. In contrast, Oct4-DECAI cells lost Oct4 expression, and consequently, SSEA4 (Fig. 5A and B), Nanog and Sox2 expression (Fig. 5C). They also changed their morphology quite dramatically (Supplementary Figure 4), indicating that these cells had lost their pluripotency. The induced knockout of Oct4 resulted in differentiation of human iPSCs with a significant increase in transcription of Cdx2 and Eomes (Fig. 5C), genes associated with trophoblast and endoderm lineages^15,16^. In line with these observations, we could detect Cre-mediated recombination at the genomic DNA level (Supplementary Figure 5). Altogether, this highlights the feasibility of our approach for conditional gene inactivation and suggests its applicability in more physiological settings.

## Discussion

In this study, we created a novel small cassette that readily allows the tagging of endogenous genes, poising them for conditional gene inactivation. The system is robust, triggers a profound decrease in gene expression and may be the first approach that can be truly applied at a large scale.

Of note, the work presented here has been inspired by the CRISPR-FLIP approach^10^. Yet we feel it goes beyond this approach in at least two key aspects: (i) gene expression is disrupted by a series of stop codons, not by revealing a splice acceptor that traps the transcript; (ii) most importantly, the cassette size is considerably smaller (201 bp versus at least 1,931 bp in CRISPR-FLIP) and hence, donor assembly is more straightforward. Additionally, even strong splice acceptors can be leaky depending on the genomic context^17^ and hence, the impact of the CRISPR-FLIP cassette may vary from gene to gene.

Experiments presented here were performed in HAP1 cells and human iPSCs, but we are confident that this approach can be applied to any mammalian cell line. While engineering in haploid cell lines, such as HAP1, is straightforward with only a single allele to target, most cell lines are diploid like iPSCs or aneuploid, harbouring multiple chromosome copies. To obtain complete conditional knockouts in such cells, we envisage two possible solutions: (i) complete bi-allelic or multi-allelic knock-in: one obtains a cell line in which both alleles of a given gene of interest have been tagged with the cassette. While this is possible, the efficiency will likely not be very high as bi-allelic tagging is infrequent. (ii) Mono-allelic knock-in with disruption of the open reading frame of the additional alleles: as a compromise, we suggest to screen for clones in which one allele has been tagged and the other allele(s) have been inactivated by Cas9 cleavage following NHEJ repair. Since the donor template is integrated in the middle of a coding exon near the start of a gene, the gRNA used to trigger incorporation of the donor will likely lead to indels abrogating gene expression. This is exemplified by our results in iPSCs where clones were recovered with both bi-allelic targeting and mono-allelic knock-in with indels on the second allele. While it may not be possible to obtain such clones in all instances as some genes may require all alleles to be intact, this is likely to represent a tolerable compromise for most genes.

The artificial intron approach presented here also has some conceivable shortcomings. First, there is a potential risk that insertion of the artificial intron may dysregulate endogenous splicing events, leading to constitutive gene inactivation. When conducting these studies, we noted one case where insertion of the artificial intron into the CDK4 gene (which is non-essential in HAP1 cells) abrogated CDK4 expression (data not shown). A more detailed analysis of these cells revealed that the intron was successfully removed by splicing, but some erroneous splicing events led to the assembly of a non-functional mRNA that was most likely degraded by non-sense mediated decay. Hence, when targeting non-essential genes, one may need to monitor the impact of cassette integration on endogenous gene expression. Fortunately, this can easily be assessed by a conventional RT-PCR. Of note, this is less of a problem for essential genes because cells bearing such editing events will not be viable.

Second, our method depends on robust Cre recombinase delivery and expression for activating the cassette. Here, we introduced Cre-ERT2 by retroviral transduction for METTL16-DECAI and OCT4-DECAI activation. While this is possible, we noticed some shortcomings, mainly: (i) Cre-ERT2 expression can be leaky and already occur in the absence of 4-OHT (data not shown) and (ii) Cre-ERT2 expression can be silenced, especially when Cre-ERT2 is delivered via retroviral infection. A more elegant approach would be to express Cre-ERT2 from a safe-harbour locus such as AAVS1 or ROSA26. This would not only be better defined from a genetic point of view, but also likely to yield more robust and reproducible levels of Cre expression.

While we feel that the method presented here offers an easy and efficient strategy for generating conditional knockouts, some alternative methods exists and are worth mentioning. Recently, modified versions of the CRISPR/Cas9 system have been adopted to modulate gene expression. One example is CRISPR interference (CRISPRi), where a catalytically dead Cas9 directed by a guide RNA will suppress expression of the target gene by sterically hindering transcription initiation/elongation or by the action of a silencing effector domain fused to Cas9^18,19^. While this strategy has the advantage of being reversible, it is difficult to reach complete inhibition of the target gene expression. Another method utilizes inducible CRISPR systems. There are several iterations of these systems ranging from split Cas9 molecules that form a catalytically active molecule upon a certain stimulus^20^ or control of Cas9 or gRNA expression by inducible promoters^21^. The efficiency of these methods will depend on the expression level of the gRNA and will likely show a variety of effects across cells in the sample since the editing induced by Cas9 will not happen in a predictable manner. This contrasts with the DECAI method which will lead to a predictable modification and disruption of expression upon Cre recombination.

Another alternative to the genetic approach presented here is a biochemical approach such as degron tagging. Degrons are drug-regulatable domains that allow one to modulate the abundance of the degron tagged protein by adding or withdrawing a small molecule. Popular degrons include the auxin/mAID system^22^, the HaloPROTAC3/ HaloTag system^23^ and the HCV protease inhibitor/ SMAShTag system^24^. At least some of the degrons that are commonly used (such as the mAID system) are much faster in degrading the client protein (with a half life of minutes^22^ rather than hours or days) as they lead to the recruitment of E3 ligases that actively degrade the protein population that is present. Additionally, the degron tags have the advantage of allowing reversible and tuneable degradation of the target proteins. However, the use of degrons has two major limitations: (i) the bi-allelic tagging of genes occurs at very low frequency and thus, it is difficult to obtain a cell line or an organism in which all copies of a gene have been conditionally inactivated and (ii) the insertion of foreign sequences within the coding region of a gene could disturb protein folding or protein function. As a consequence, degron tagging can be cumbersome and may not be compatible with high-throughput applications.

With this in mind, we feel that the approach we devised is more suitable for serial production of conditional knockouts, whereas degron tagging may be the method of choice if the protein is well understood and inefficient clone recovery is less of a constraint.

In summary, the DECAI method presented here offers a convenient and efficient alternative for generating conditional knockout alleles in mammalian cells.

## Electronic supplementary material

Supplementary Figures
