# Supplementary material for: A small cassette enables conditional gene inactivation by CRISPR/Cas9
Source: Sci Rep. 2017 Dec 1;7:16770. doi: 10.1038/s41598-017-16931-z (PMC5711947; doi:10.1038/s41598-017-16931-z)
Supplement: Supplementary file 1 — Supplementary Figures [file 41598_2017_16931_MOESM1_ESM.pdf]

Supplementary figures for “A small cassette enables conditional gene inactivation by CRISPR/Cas9”

Paloma M Guzzardo[1*], Christina Rashkova[1*], Rodrigo L dos Santos[2*], Raha Tehrani[2], Philippe Collin[2] and Tilmann Bürckstümmer[1]

1. Horizon Genomics GmbH, Vienna, Austria
2. Horizon Discovery Ltd, Cambridge, UK

* These authors contributed equally

Corresponding Author:

Dr. Tilmann Bürckstümmer

Campus Vienna Biocenter 3

1030 Wien

Austria

Email t.buerckstuemmer@horizondiscovery.com

CD46-DECAI donor sequence:

TTACCACCACCACCACCCCCCTGCAGTAGTTTGGTAAAAACTACCAAACAAAATTTTAAAAAGCAGCCAGTGAGTGGGGAAAACAACAAAAACCCGTATTACATTTAGGAGG
GCAGGGACAAGTAAGAATGACAGCCAGCCAAGTTTTCTTAAGAAAATGTGGTTACAATATGGTTATCTCTGTTAGTGCTGAAAGAAAAATAAAACACCCTAGAATTCTGTATG
AAGGAGAATATATTAGTTAACATTTAAAGTCTTTTAAAAAATGTTTTTAACATCTTGCATTCCATTCCTTGTCTCTGTTCACACTGGAAATTACTACTTTGTACTACTTTTTCTGCT
AAAGCAGATATCCATTAATTCTGAGGTTTCTCTAATTTTCCAGTGGTCAAATGTCGATTTCCAGTAGTCGAAAATGGAAAACAGATATCAGGATTTGGAAAAAAATTTTACTAC
AAAGCAACAGTTATGTTTGAATGCGATAAGGTAAGTAATAACTTCGTATAGCATACATTATACGAAGTTATTCAAGGTTAGAAGACAGGTTTAAGGAGACCAATAGAAACTGG
GCTTGTCGAGACAGAGAAGACTCTTGCGTTTCTGATAGGCACCTATTGGTCTTACTGACATCCACTTTGCCATAACTTCGTATAGCATACATTATACGAAGTTATTTTCTCTCCAC
AGGGTTTTTACCTCGATGGCAGCGACACAATTGTCTGTGACAGTAACAGTACTTGGGATCCCCCAGTTCCAAAGTGTCTTAAAGGTACAAAGGTTATCTTTTTTCTGTCTTGG
TTTGTTATTGTTGTTGCTGTTCATTTTAGACTTTATTTCTTTGATATTAACTATCAGTCATACAAAATAACTGAAAAGAAACAATTTTAGTATTTAACTCTGTCTTGTATTCATTTCT
ATGCCAGATGAATGACACGAAATTCACATAAAATTCTGCTGTTGTGATTTTTTGTGCTTTTCCAGGGTTCTTAGCACGTTATGTACATTGCATGGGTATATGCTTTTAATATTTTT
ATGTATAAAAAGTGAATTACAACAACTTTTTGGAATTGAAACATGGGCATTTTTATCTAAGTAAGTCAACAATGGCATAATTCATATAAATGAAATGAGAGCAATAACTCCCAAG
TGGTTGATCTTCTAACATTATTTTGTTTCCTAGTGCTGCCTCCATCTAGTACAAAACCTCCAGCTTTGAGTCATTCAGGTTTAGTAGCTTCTTCCTTATATGTCTTCTTCCTTATATG
TTACAA

METTL16-DECAI donor sequence:

ACTTGTTCAAAGTCACACAGTGAGGAGACGACGAGACAGGAATTTGAGCCGAGTCATTTAATCTCTGTGCTCCATATACGGATATGCAGTGTTTTTGGTGGAGGATTGTACTG
ATTTTCATAAGCTGTGACTGTGTGAAACACTCGTGTATGTGCCACTGGGATGAGTGCTCTGTGTAACTGTGTGTGATCTCCCTGGGAGGTTCGGATGGGTATTTGGTTCTGCC
TGTTTGCCGTAGAGCTTGTACAACATAGTGAATCTTACTAATCAGTTTTACTTCCTTTTCTTTATTCCTCAGCCTTAATTTTAAAGACCCCGAAGCAGTCAGAGCTCTGACGTGT
ACTCTCCTAAGGTAAGTAATAACTTCGTATAGCATACATTATACGAAGTTATTCAAGGTTAGAAGACAGGTTTAAGGAGACCAATAGAAACTGGGCTTGTCGAGACAGAGAAG
ACTCTTGCGTTTCTGATAGGCACCTATTGGTCTTACTGACATCCACTTTGCCATAACTTCGTATAGCATACATTATACGAAGTTATTTTCTCTCCACAGGGAAGATTTTGGACTTT
CTATTGATATTCCATTGGAGAGACTAATTCCCACAGTTCCCTTGAGACTCAACTATATTCACTGGGTAGAAGATCTGATCGGTCACCAGGATTCTGACAAAAGTACTCTCCGAA
GAGGAATTGACATAGGTATATCATTTTAAATTCTTTTTTGGCTAAACAGTTTTCATAAGTTTTGCGAGATCAAATTCTTTGTAAACTTTTCTACTGGGTATTTGTTGTTGTACAAA
AGAAGCTGGCTACAGATACAGGTTGAGCATCCTTAATCCGAAAATCCGAAATGCTTCAAAATCTGCAACCTTTGAGCACTGATGTGTTGCTCAAAGGCAATGTGCGTTGGAG
CGTTCCAGATTTGGGGGTTAGGGATGTTCAGCCTGTAAATATAGTGCACATATTTCAAAATCTGAAAAAAAAATTGAAATCCAAAACA

Oct4-DECAI#1 donor sequence:

CCTTCGCCTCAGTTTCTCCCCCCACCTCCCTCTCCTCCACCCATCCAGGGGGCGGGGCCAGAGGTCAAGGCTAGTGGGTGGGACTGGGGAGGGAGAGAGGGGTTGAGTA
GTCCCTTCGCAAGCCCTCATTTCACCAGGCCCCCGGCTTGGGGCGCCTTCCTTCCCCATGGCGGGACACCTGGCTTCGGATTTCGCCTTCTCGCCCCCTCCAGGTGGTGGAG
GTGATGGGCCAGGGGGGCCGGAGCCGGGCTGGGTTGATCCTCGGACCTGGCTAAGCTTCCAAGGCCCTCCTGGAGGGCCAGGAATCGGGCCGGGGGTTGGGCCAGGCT
CTGAGGTGTGGGGGATTCCCCCATGCCCCCCGCCGTATGAGTTCTGTGGGGGGATGGCGTACTGTGGGCCCCAGGTAAGTAATAACTTCGTATAGCATACATTATACGAAGTT
ATTCAAGGTTAGAAGACAGGTTTAAGGAGACCAATAGAAACTGGGCTTGTCGAGACAGAGAAGACTCTTGCGTTTCTGATAGGCACCTATTGGTCTTACTGACATCCACTTT
GCCATAACTTCGTATAGCATACATTATACGAAGTTATTTTCTCTCCACAGGTTGGAGTGGGGCTAGTGCCCCAAGGCGGCTTGGAGACCTCTCAGCCTGAGGGCGAAGCAGG
AGTCGGGGTGGAGAGCAACTCCGATGGGGCCTCCCCGGAGCCCTGCACCGTCACCCCTGGTGCCGTGAAGCTGGAGAAGGAGAAGCTGGAGCAAAACCCGGAGGAGGC
AAGTGAGCTTCGACGGGGTTGGGGTGTGGGGAGGTGGTCATGACAGGGCAGCCTGATGGGGAAGTGGTCACCTGCAGCTGCCCAGACCTGGCACCCAGGAGAGGAGCA
GGCAGGGTCAGCTGCCCTGGCCAGGGAGGGGTGTGTATCAACTGCTGGCAGCCCTGGCAGGCAGGGGCCAGGTGGGAAGTGGAAGCTGGATTTCGAAGAGACAACTGC
CGGTGAGGGCAGAGC

Oct4-DECAI#2 donor sequence:

GAGGCTACATTGAGCCATCATTGTACTCCACTGCACTCCAGTCTGGGCAACAAAGTGAGACCCTGTCTTAAAAAATAAAAATAAAAAAAGTTTCTGTGGGGGACCTGCACTG
AGGTCCTGGAGGGGCGCCAGTTGTGTCTCCCGGTTTTCCCCTTCCACAGACACCATTGCCACCACCATTAGGCAAACATCCTTCGCCTCAGTTTCTCCCCCCACCTCCCTCTCC
TCCACCCATCCAGGGGGCGGGGCCAGAGGTCAAGGCTAGTGGGTGGGACTGGGGAGGGAGAGAGGGGTTGAGTAGTCCCTTCGCAAGCCCTCATTTCACCAGGCCCCCG
GCTTGGGGCGCCTTCCTTCCCCATGGCGGGACACCTGGCTTCGGATTTCGCCTTCTCGCCCCCTCCAGGTAAGTAATAACTTCGTATAGCATACATTATACGAAGTTATTCAAG
GTTAGAAGACAGGTTTAAGGAGACCAATAGAAACTGGGCTTGTCGAGACAGAGAAGACTCTTGCGTTTCTGATAGGCACCTATTGGTCTTACTGACATCCACTTTGCCATAA
CTTCGTATAGCATACATTATACGAAGTTATTTTCTCTCCACAGGTGGTGGAGGTGATGGGCCAGGGGGGCCGGAGCCGGGCTGGGTTGATCCTCGGACCTGGCTAAGCTTCC
AAGGCCCTCCTGGAGGGCCAGGAATCGGGCCGGGGGTTGGGCCAGGCTCTGAGGTGTGGGGGATTCCCCCATGCCCCCCGCCGTATGAGTTCTGTGGGGGGATGGCGTA
CTGTGGGCCCCAGGTTGGAGTGGGGCTAGTGCCCCAAGGCGGCTTGGAGACCTCTCAGCCTGAGGGCGAAGCAGGAGTCGGGGTGGAGAGCAACTCCGATGGGGCCTC
CCCGGAGCCCTGCACCGTCACCCCTGGTGCCGTGAAGCTGGAGAAGGAGAAGCTGGAGCAAAACCCGGAGGAGGCAAGTGAGCTTCGACGGGGTTGGGGTGTGGGGA
GGTGGTCA

Supplementary Figure 1. Homology donor templates for cell line generation. Homology donor templates used to engineer the CD46-DECAI, METTL16-DECAI and OCT4-DECAI cell lines are shown. The donors contain ~400bp right and left homology arms flanking the 201bp artificial intron cassette (underlined).

Supplementary Figure 1

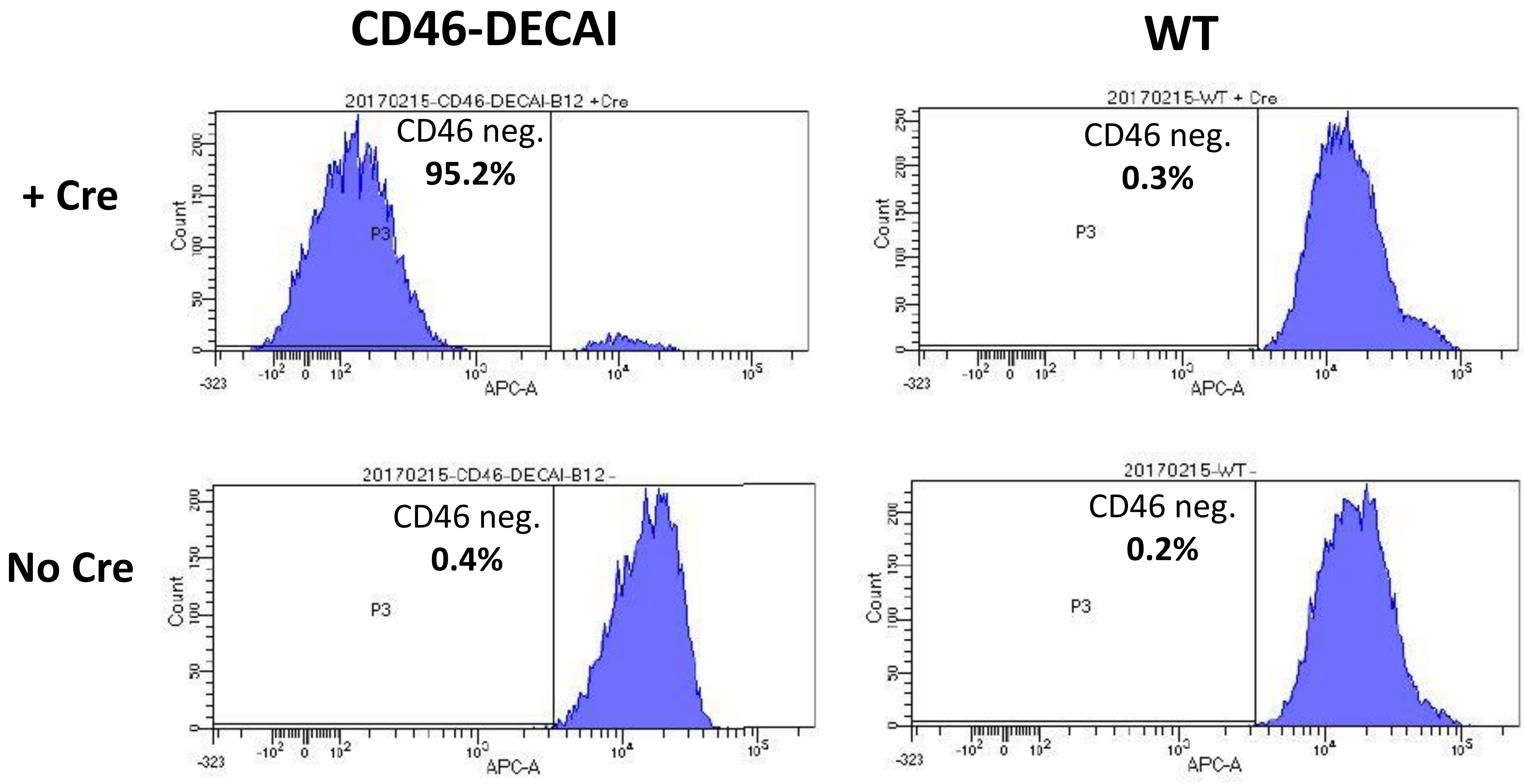

Supplementary Figure 2. Analysis of CD46 expression in CD46-DECAI and WT Hap1 cells. HAP1 wild-type and CD46-DECAI cells were transfected with a plasmid expressing Cre recombinase. Cells were then stained with a CD46-specific antibody and analysed by flow cytometry.

Supplementary Figure 2

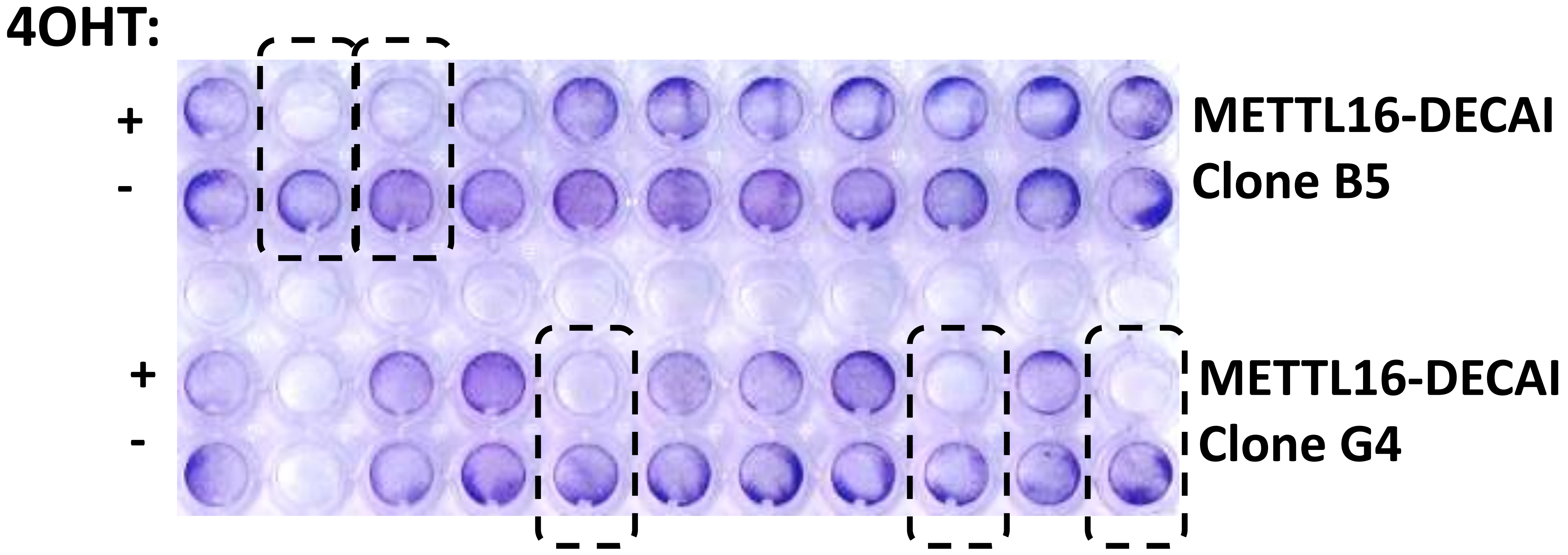

Supplementary Figure 3. Isolation of Cre-ERT2 expressing METTL16-DECAI clones

Crystal violet staining of single cell clones isolated from two METTL16-DECAI cell lines (Clone B5 and Clone G4). 12 clones of each cell line were isolated following transduction with Cre-ERT2 and these potentially Cre-ERT2 expressing clones were treated with 4-OHT to trigger cassette activation. Clones in which 4-OHT triggered cassette activation, and hence, cell death, are marked with a box.

Supplementary Figure 3

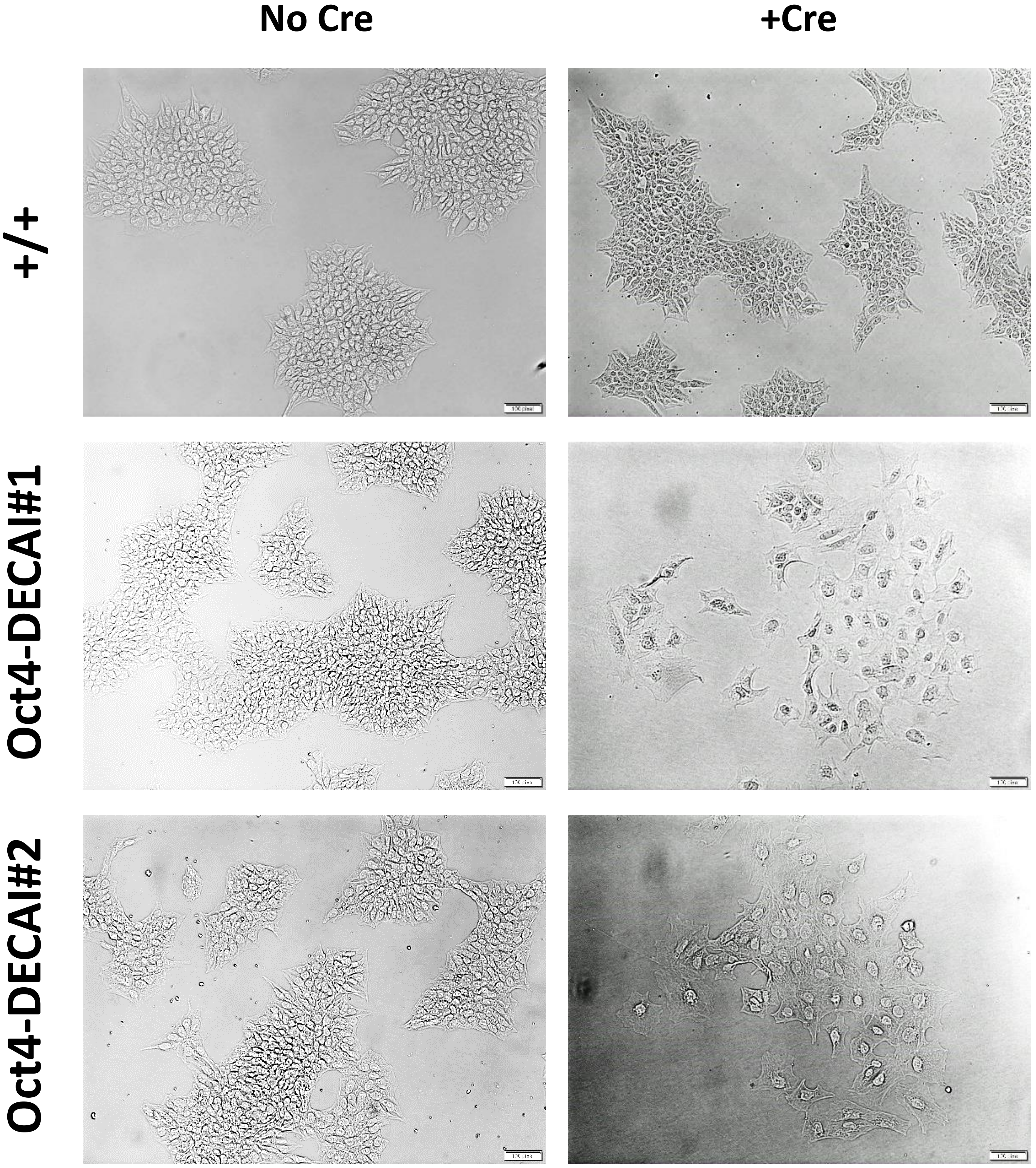

Supplementary Figure 4. Cre-recombination leads to a change in morphology of Oct4-DECAI cells. Bright field microscopy images of WT, Oct4-DECAI#1 and Oct4-DECAI#2 after transfection with Cre-ERT2 and 4-OHT treatment. A clear change in cell morphology is observed only in the Oct4-DECAI#1 and Oct4-DECAI#2 cells.

Supplementary Figure 4

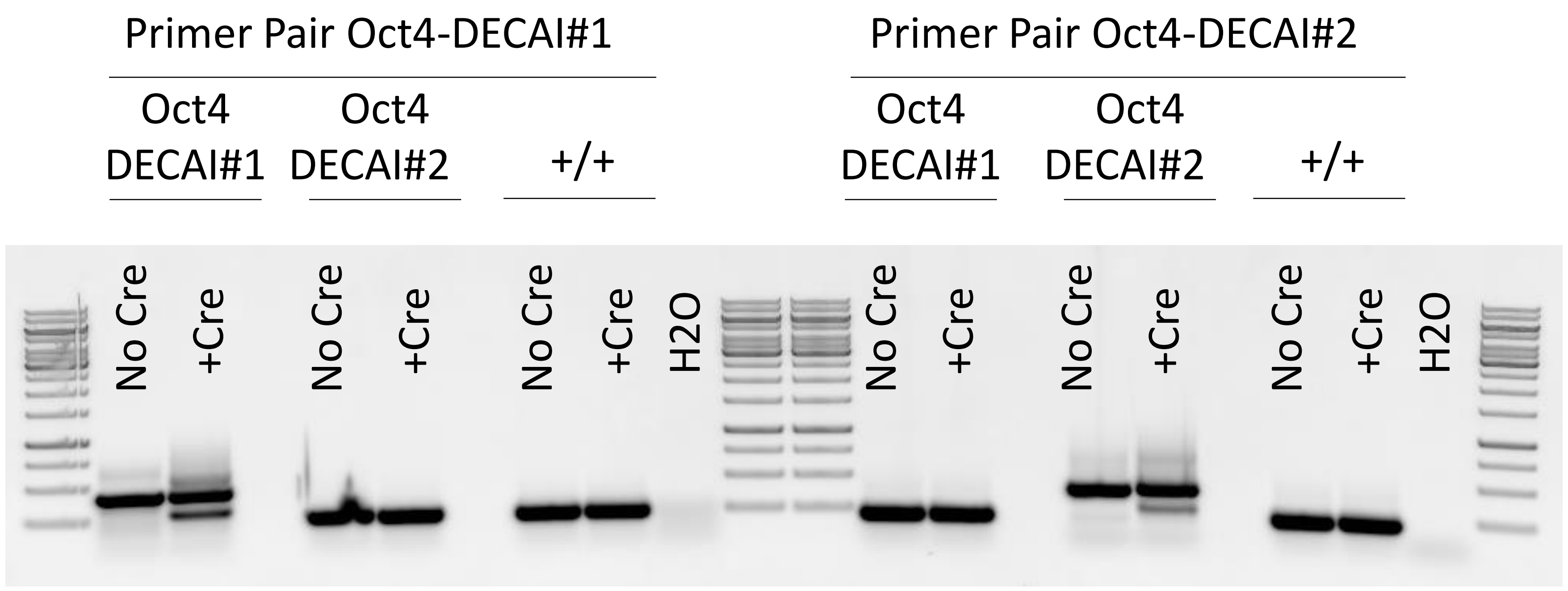

Supplementary Figure 5. Cre-recombination leads to branch point excision in Oct4-DECAI cells. Genomic DNA was isolated from WT, Oct4-DECAI#1 and Oct4-DECAI#2 cells that had been transfected with Cre-ERT2 and treated with 4-OHT, and from cells not transfected with Cre-ERT2. Samples were genotyped using PCR primers specific to either the cassette insertion site of Oct4-DECAI#1 or Oct4-DECAI#2.

Supplementary Figure 5
